# Nursing Practices for Dyspnea Management in Patients with Cancer Based on Monthly and Weekly Prognoses: A Multi-Site Cross-Sectional Study of Palliative Care Nurses in Japan

**DOI:** 10.1089/pmr.2024.0058

**Published:** 2024-10-14

**Authors:** Jun Kako, Miharu Morikawa, Kohei Kajiwara, Masamitsu Kobayashi, Yusuke Kanno, Kimiko Nakano, Yoshinobu Matsuda

**Affiliations:** ^1^ Graduate School of Medicine, Mie University, Tsu, Japan.; ^2^ Graduate School of Medicine, Kyoto University, Kyoto, Japan.; ^3^ Japanese Red Cross Kyushu International College of Nursing, Munakata, Japan.; ^4^ Graduate of Nursing Science, St. Luke’s International University, Tokyo, Japan.; ^5^ Nursing Science, Tokyo Medical and Dental University, Tokyo, Japan.; ^6^ Mie University Hospital, Tsu, Japan.; ^7^ Department of Psychosomatic Internal Medicine, NHO Kinki Chuo Chest Medical Center, Sakai, Japan.

**Keywords:** cancer, cross-sectional studies, dyspnea, nursing practice, palliative care

## Abstract

**Background and Purpose:**

Dyspnea in patients with terminal cancer worsens near death, necessitating effective nonpharmacological management. Despite guideline recommendations, detailed studies on nonpharmacological nursing practices are scarce. This study aimed to elucidate nursing practices for dyspnea in patients with cancer based on monthly and weekly prognoses.

**Methods:**

A multi-site cross-sectional study was conducted among nurses in 389 palliative care units in Japan. The study surveyed the frequency of direct care practices for dyspnea management (nurse-led intervention, multidisciplinary intervention, psychoeducational programs, breathing techniques, walking therapy, inspiratory muscle training, respiratory rehabilitation, yoga, acupressure, fan therapy, guided imagery, abdominal massage, aromatherapy, and a reduction in room temperature and humidity) in patients with cancer with monthly and weekly prognoses.

**Results:**

Of the 389 invited units, 162 participated. From these, 2448 registered nurses were invited and 539 (22.3%) responded. Almost similar nursing practices were provided regardless of patient prognosis. Nurse-led intervention was the most frequently practiced, followed by room temperature and humidity reduction, multidisciplinary intervention, and fan therapy. Yoga, respiratory rehabilitation, and acupressure were rarely practiced.

**Conclusion:**

Nursing practices for dyspnea are similar, irrespective of prognosis. Nurse-led interventions, reducing room temperature and humidity, multidisciplinary intervention, and fan therapy are frequently used for dyspnea in patients with cancer. Future studies should evaluate the effectiveness of these nursing practices.

## Introduction

Dyspnea in patients with terminal cancer is associated with anxiety, depression, and decreased motivation to live, significantly affecting their quality of life and activities of daily living.^1–6^ The frequency and severity of dyspnea tend to worsen as death approaches.^7,8^ The effective management of this symptom is challenging and often requires symptomatic treatment as the primary approach. Clinical guidelines recommend nonpharmacological interventions as initial strategies due to their safety and high convenience,^9,10^ particularly in the context of different prognostic timeframes.^11^ However, nonpharmacological interventions specific to monthly and weekly prognoses in patients with cancer remain understudied.

Nurses play a crucial role in delivering nonpharmacological interventions to terminally ill patients with cancer. The guidelines indicate that the support provided depends on the prognosis based on the patient’s vulnerability.^9,10^ A previous study reported nursing support for dyspnea in patients with cancer;^12^ however, specific nursing practices in terms of monthly and weekly prognoses remain unclear, and there is a lack of evidence regarding nursing support for dyspnea in patients with terminal cancer. Hence, this novel study aimed to elucidate nursing practices for dyspnea in patients with cancer based on monthly and weekly prognoses among nurses working in palliative care units in Japan. The findings are expected to reveal the prioritization and efficacy assessments of nursing practice in terminal cancer, an area lacking sufficient evidence.

## Methods

### Study design

This multi-site, cross-sectional study included nurses from palliative care units across Japan. This study was approved by the Clinical Research Ethics Review Committee of our University Hospital (U2023-011) and registered with the University Hospital Medical Information Network, Japan (UMIN0000 52329). All the participants provided written informed consent. The reporting followed the Strengthening the Reporting of Observational Studies in Epidemiology (STROBE) guidelines.^13^

### Setting

Specialized palliative care in Japan encompasses palliative care units and teams.^14^ This survey specifically targeted registered nurses working in these specialized palliative care units. Participants were recruited from all 389 palliative care units across Japan (as of 14 September 2023) that had been accepted for notification of admission charges for palliative care units.

### Participants and study procedure

The inclusion criterion for nurses was provision of direct care in palliative care units. The exclusion criteria were as follows: (1) manager of a palliative care unit (e.g., head nurse), (2) nurses who came to the palliative care unit for training from other wards, and (3) nurses who came to the palliative care unit for training from other hospitals.

All eligible facilities were invited to participate in this study. Subsequently, the facilities that provided consent were sent an explanatory letter, granted individual access to an online questionnaire regarding the number of nurses in the palliative care unit, and requested to participate in the survey. Only nurses who agreed to participate in the study completed the online questionnaire. The data for this study were collected from October 1, 2023, to March 31, 2024, via online surveys conducted using LimeSurvey Cloud offered by LimeSurvey GmbH (cf. https://www.limesurvey.org/ja).

### Measurements

The online survey enquired about the nursing practice for dyspnea in patients with cancer based on monthly and weekly prognoses. The nursing practices comprised findings from a scoping review of nursing practices for dyspnea in patients with cancer,^12^ along with nursing support acquired through interviews with nurses in a palliative care unit. Fourteen nursing supports were identified, including nurse-led intervention (assessment of dyspnea, evaluation after implementation of care, provision of information, and instruction on breathing techniques and management methods), multidisciplinary intervention (nurse managed assessment, information provision, direct care, and coordination with other professionals, while other professionals provide pharmacotherapy and counseling as needed), psychoeducational programs (relaxation, activity scheduling, and psychoeducation in collaboration with psychologists), breathing techniques, walking therapy, inspiratory muscle training, respiratory rehabilitation, yoga, acupressure, fan therapy, guided imagery, abdominal massage, aromatherapy, and reduction in room temperature and humidity. These nursing supports were evaluated in terms of the status of nursing practice for patients with cancer based on monthly and weekly prognoses using a 5-point Likert scale (1 = not at all; 2 = rarely; 3 = occasionally; 4 = frequently; and 5 = very frequently). In addition, age, sex, years of nursing experience, years of experience in the palliative care unit, educational background, and qualifications were collected as background information from the participants.

### Statistical analyses

Descriptive statistics were used to analyze the demographic information and nursing practice frequencies. EZR, a component of R software, was used for statistical analysis.^15^ Additionally, data with missing responses were not included in the analysis.

## Results

### Characteristics of participants

A total of 389 palliative care units in Japan were invited to participate in the study, and consent was obtained from 162 facilities. A total of 2448 registered nurses working in palliative care units across 162 facilities were invited to participate in the questionnaire survey and 539 (22.3%) responded. The average age of the respondents was 42.3 ± 9.8 years, and more than 90% were women. The average number of years of nursing experience was approximately 19, and the average number of years in the palliative care unit was approximately 5. Less than 10% of the respondents held certifications for palliative care (Table 1).

**Table 1.** Characteristics of Participants (*n* = 539)

Characteristics *n* (%) or mean (SD)	
Age (years)	42.3 (9.8)
Sex	
Female	511 (94.8)
Male	26 (4.8)
Prefer not to answer	2 (0.4)
Years of nursing experience	18.6 (9.6)
Years of experience in palliative care unit	4.8 (4.3)
Educational background	
Nursing vocational school (including junior college)	456 (84.6)
Nursing university	64 (11.9)
Master’s program (pre-doctoral program)	10 (1.9)
Doctoral program (post-doctoral program)	1 (0.2)
Qualifications	
Certified nurse in palliative care	29 (5.3)
Certified nurse in cancer pain management nursing	4 (0.7)
Certified nurse specialist in cancer nursing	3 (0.6)
Qualifications (Other)	
End-of-life care specialist	8 (1.5)
Certified public psychologist	2 (0.4)
Others	6 (1.1)

### Nursing practice for dyspnea in patients with cancer (prognosis: months)

The most commonly practiced interventions for dyspnea include nurse-led interventions, followed by temperature and humidity reduction, multidisciplinary approaches, and fan therapy. However, yoga, respiratory rehabilitation, acupressure, and inspiratory muscle training were rarely implemented (Table 2).

**Table 2.** Nursing Practice for Dyspnea in Patients with Cancer Based on Monthly and Weekly Prognoses (*n* = 539)

Nursing practice	Prognosis*, mean (SD)	
	Months	Weeks
Nurse-led intervention	3.46 (1.06)	3.52 (1.10)
Reduce room temperature and humidity	3.38 (1.05)	3.51 (1.06)
Multidisciplinary intervention	3.26 (1.13)	3.33 (1.16)
Fan therapy	2.87 (1.24)	3.07 (1.30)
Breathing technique	2.59 (0.98)	2.53 (1.03)
Psychoeducational program	2.19 (1.14)	2.19 (1.17)
Guided imagery	2.10 (1.11)	2.15 (1.15)
Aromatherapy	1.97 (1.06)	2.02 (1.10)
Walking therapy	1.78 (0.92)	1.59 (0.82)
Abdominal massage	1.71 (0.93)	1.73 (0.93)
Inspiratory muscle training	1.31 (0.68)	1.26 (0.66)
Acupressure	1.24 (0.66)	1.25 (0.67)
Respiratory rehabilitation	1.20 (0.58)	1.17 (0.55)
Yoga	1.11 (0.48)	1.10 (0.46)

^*^ 5-point Likert scale (1 = not at all; 2 = rarely; 3 = occasionally; 4 = frequently; and 5 = very frequently).

### Nursing practice for dyspnea in patients with cancer (prognosis: weeks)

Weekly prognoses nursing practices mirrored those for monthly prognoses, with abdominal massage and walking therapy as the only difference. (Table 2)

## Discussion

### Main findings

This study investigated nursing practices for managing dyspnea in patients with cancer in Japanese palliative care units, focusing on monthly and weekly prognoses. The results showed that almost the same nursing practice was provided, regardless of the prognosis of patients. Although guidelines indicate that the support provided may be limited by the prognosis of patients and their condition,^9^ the results of this study did not support this. The precise reasons for this observation remain unclear; however, resource constraints could potentially play a role and warrant further investigation in future studies.

The palliative care unit nurses frequently implement nurse-led multidisciplinary interventions. These interventions combine multiple supports rather than a single approach and are expected to enhance the management of dyspnea. Given that dyspnea is influenced by various factors, assessing it and providing support from multiple perspectives is crucial.^16^ The clinical practice guidelines of the European Respiratory Society recommend a comprehensive assessment and identification of needs as the initial approach to dyspnea in patients with serious respiratory illnesses, followed by a multi-component intervention.^17^ Despite being a long-term strategy, multi-component interventions can be administered regardless of patient prognosis; however, future studies are needed to evaluate the effectiveness of multi-component interventions, specifically in terminal cancer care.

The implementation frequencies of yoga, respiratory rehabilitation, inspiratory muscle training, and walking therapy were low, as expected. A previous study examining the applicability of the Delphi method to patients with terminal cancer noted issues of invasiveness, compatibility, and perceived effectiveness within prognosis weeks,^18^ resulting in the low applicability of these interventions. Thus, the findings of our study support those of the previous study. Conversely, the same previous study indicated the high applicability of acupressure,^18^ despite its low implementation frequency. In contrast to previous findings, challenges related to invasiveness and compatibility were minimal for acupressure in our study. These discrepancies may stem from the varying access to knowledge and information. Hence, future research should focus on validating the effectiveness of acupressure interventions and on enhancing nurses’ knowledge and skills in this area.

Fan therapy was implemented less frequently than anticipated, despite being an applicable support regardless of prognosis.^19,20^ Clinical practice guidelines recommend fan therapy as the nonpharmacological intervention with the highest level of evidence.^9,10^ Evidence supporting the effectiveness of fan therapy in patients with terminal cancer has accumulated over the past decade, suggesting its underutilization in clinical practice. Therefore, future efforts to disseminate and implement fan therapy are needed. Conversely, reductions in room temperature and humidity were implemented more frequently than expected. Although no studies have reported its effectiveness, it is widely believed to be effective based on clinical expertise. Cold stimulation of the cheeks has been noted to be effective in alleviating dyspnea.^21,22^ Therefore, reducing room temperature and humidity may effectively lower patients’ perceived temperature. However, further research is required to elucidate this mechanism.

### Strengths and limitations

The strengths included a comprehensive evaluation of nursing practices for dyspnea in patients with terminal cancer across different prognoses. However, low participation rates and potential cultural influences on practice may limit its generalizability. In addition, the reported frequency of practice may not fully reflect actual implementation. Despite these limitations, this study offers valuable insights for future research and clinical practice.

## Conclusions

This study revealed that nurses in Japanese palliative care units provided similar support for dyspnea in patients with terminal cancer on a prognostic monthly and weekly basis. Nurses most frequently practiced nurse-led interventions, followed by reduced room temperature and humidity, multidisciplinary interventions, and fan therapy. Future research should evaluate the effectiveness of nursing practices more frequently.

## Data Availability

The data were registered in the Individual Case Data Repository of the UMIN (University Hospital Medical Information Network), Japan.
